# Quality and storage characteristics of yogurt containing *Lacobacillus sakei* ALI033 and cinnamon ethanol extract

**DOI:** 10.1186/s40781-016-0098-0

**Published:** 2016-04-25

**Authors:** Yu Jin Choi, Hee Yeon Jin, Hee Sun Yang, Sang Cheon Lee, Chang Ki Huh

**Affiliations:** Imsil Research Institute of Cheese Science, 50 Doin 2-gil, Seongsu-myeon, Imsil-gun, Jeollabuk-do 55918 South Korea

**Keywords:** Antifungal-active lactic acid bacteria, Cinnamon extract, Antifungal activity, Yogurt, Quality and storage characteristics

## Abstract

**Background:**

This study was conducted to examine the quality and storage characteristics of yogurt containing antifungal-active lactic acid bacteria (ALH, *Lacobacillus sakei* ALI033) isolated from kimchi and cinnamon ethanol extract. The starter was used for culture inoculation (1.0 % commercial starter culture YF-L812 and ALH).

**Results:**

The antifungal activity of cinnamon extracts was observed in treatments with either cinnamon ethanol extracts or cinnamon methanol extracts. Changes in fermented milk made with ALH and cinnamon extract during fermentation at 40 °C were as follows. The pH was 4.6 after only 6 h of fermentation. Titratable acidity values were maintained at 0.8 % in all treatment groups. Viable cell counts were maintained at 4 × 10^9^ CFU/mL in all groups except for 1.00 % cinnamon treatment. Sensory evaluations of fermented milk sample made with ALH and 0.05 % cinnamon ethanol extract were the highest. Changes in fermented milk made with ALH and cinnamon ethanol extract during storage at 4 °C for 28 days were as follows. In fermented milk containing ALH and cinnamon ethanol extracts, the changes in pH and titratable acidity were moderate and smaller compared with those of the control. Viable cell counts were maintained within a proper range of 10^8^ CFU/mL.

**Conclusions:**

The results of this study suggest that the overgrowth of fermentation strains or post acidification during storage can be effectively delayed, thereby maintaining the storage quality of yogurt products in a stable way, using cinnamon ethanol extract, which exhibits excellent antifungal and antibacterial activity, in combination with lactic acid bacteria isolated from kimchi.

## Background

The market size of fermented drinks is valued at 1600 billion won and amounts to 40 % of the total drink market, and fermented milk constitutes 81 % of the fermented drink market [1, 2], thus the market for fermented milk is expected to show considerable growth in the future. Fermentation with lactobacillus is a method of processing foods which increases the quality of foods by increasing the nutrition, generating biologically active components and improving the storage stability through the biosynthesis of antibacterial substances [3, 4]. Kimchi is a representative fermented food of our country and various microorganisms are involved in the process of fermentation of kimchi. Many studies have reported on the health functionality of kimchi lactic acid bacteria [5], and, development of diverse methods for its application is needed in order to increase their industrial value.

*Cinnamomum cassia* bark is the surface of a tall ever-green tree belonging to the Lauraceae family which grows in southern China, Vietnam, and elsewhere. *Cinnamomum cassia* bark is the outer surface of the tree, *Cinnamomum cassia* sprig is its small branch, *Cinnamomum cassia* inner bark refers to the thick inner surface, and *Cinnamomum cassia* cortex indicates the thick flattened dried bark [6]. *Cinnamomum cassia* bark is composed of 1 ~ 3.4 % essential oil, consisting of 75 ~ 90 % Cinnamic Aldehyde, 2 ~ 3 % tannin, and carbohydrates [7], which have been variously used not only as a medicine which serves as a diaphoretic, antipyretic, or analgesic, but also as a spice for foods. In addition, it is known to promote bowel peristalsis and have an antiseptic effect which suppresses hetero fermentation in intestines and serves as a natural antimicrobial which inhibits microbial contamination and growth, and research for its extensive use as a food additive, a medicine, or an industrial substance is underway [8, 9]. Park and et al. [10] investigated its antifungal effects including increasing storage period by adding them to foods, and in research by Cho and et al. [11] cinnamon extract showed excellent antifungal activity against molds and fungi including *C. albicans and A. niger.* However, no studies reporting the improvement of the storage stability of fermented milk using cinnamon extracts have been reported.

Therefore, the aim of this research is to measure the antifungal activity of cinnamon extracts, thereby suggesting a production method for fermented milk which enables manufacture of yogurt with excellent antifungal and antibacterial activity without affecting its sensory characteristics through the synergy between lactic acid bacteria (ALH, *Lacobacillus sakei* ALI033) isolated from kimchi and cinnamon ethanol extracts, and which also maintains the stability of the storage quality of yogurt by effectively delaying either the overgrowth of starter cultures or post acidification.

## Methods

### Materials

Milk (Imsil Province, Korea) was used as a raw material for yogurt preparation. Starter cultures were constituted by antifungal-active lactic acid bacteria (ALH, *Lacobacillus sakei* ALI033) isolated from kimchi [12] and commercial lactic acid bacteria (YF-L812, *Lactobacillus delbruckii* subsp. *bulgaricus* and *Streptococcus thermophilus*, Chr. Hansen, Denmark). *Penicillium brevicompactum* strain FI02 isolated from ripening cheese at the Imsil Research Institute of Cheese Science (IRICS) was used as an indicator fungus, incubated on potato dextrose agar (PDA, Difco, USA) at 30 °C for 2 days, and stored at 4 °C.

### Preparation of cinnamon extracts by different solvents

Water extract of cinnamon was homogenized to a fine powder, then 5 g of it was macerated with 100 mL of water at room temperature for 24 h. Methanol and ethanol extract were also prepared by the same method used for the water extraction process. The extracts were filtered and stored at 4 °C.

### Antifungal activity assays

Paper disc assay [13] was used for detection of antifungal activities. Plates were prepared by adding *Penicillium brevicompactum* strain FI02 culture medium (10^6^ spores per 20 mL of PDA) up to a concentration of 1.5 % (w/v). For the paper disc assay, paper discs (diameter 8 mm; Advantec, Tokyo, Japan) on PDA plates were spotted with 100 μL of cinnamon extracts using different solvents. The plates were incubated at 30 °C for 48 h and examined for inhibition zones. Antifungal activity was expressed as clear zone size (mm). The above described experiment was performed in triplicate.

### Production of cinnamon-supplemented yogurt

**Fig. 1:**
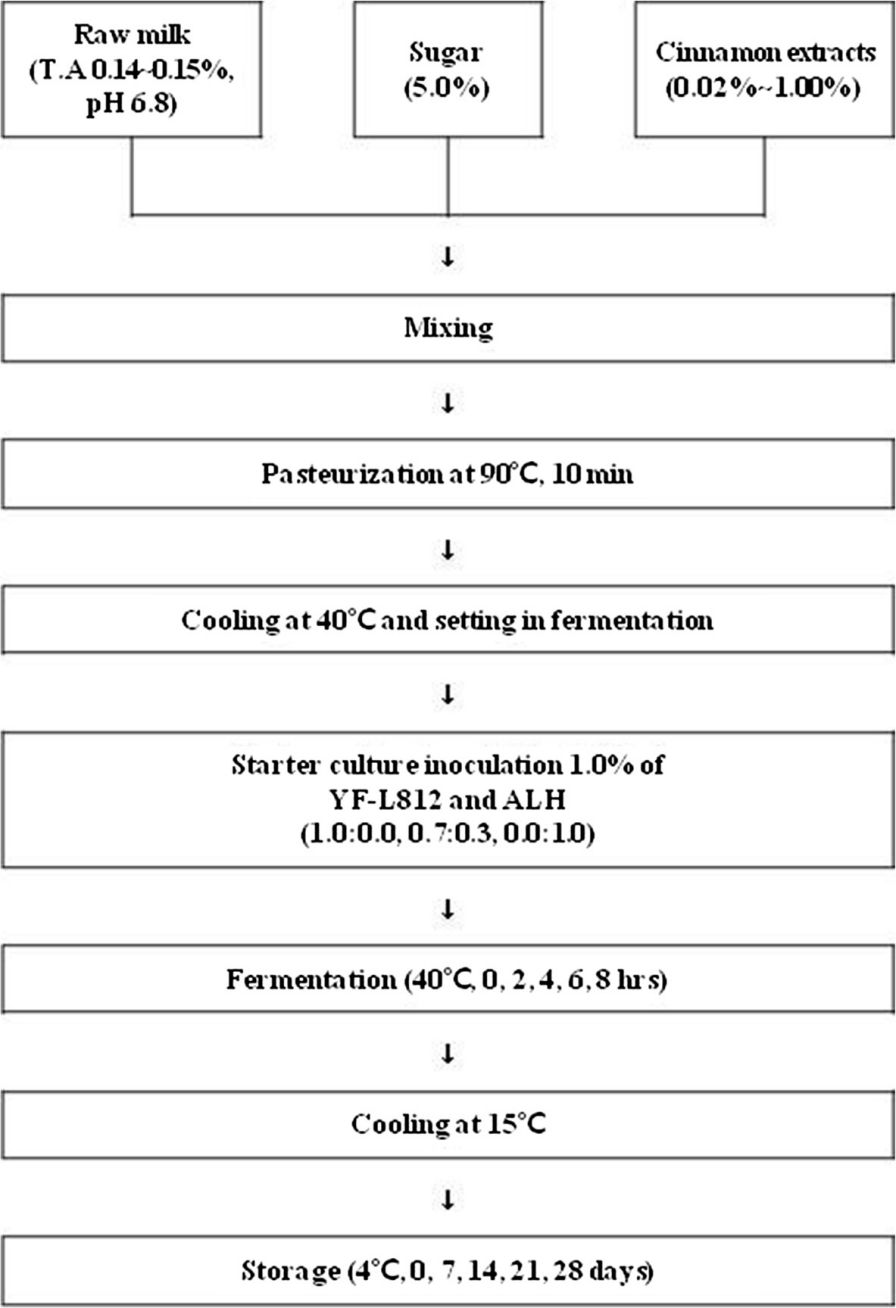
Procedures of yogurt made with lactic acid bacteria isolated from kimchi and cinnamon ethanol extract

Milk was combined with different concentrations (0.02 % ~ 1.00 %) of cinnamon ethanol extracts, and then mixed with sugar (5.0 %, w/v). The milk solution was heated at 90 °C for 10 min and cooled to 40 °C. Mixes were then inoculated with 1.0 % (v/v) starter culture (YF-L812 (Chr. Hansen, Pty. Ltd., Bayswater, Australia) and ALH; 1.0:0.0, 0.7:0.3, 0.0:1.0), and allowed to ferment at 40 °C for 8 h, and stabilized at 15 °C for 24 h [14]. After stabilization, each yogurt sample was stored for 0, 7, 14, 21, and 28 days at 4 °C in a refrigerator for evaluation of the physicochemical and sensory properties. Each batch of yogurt was made in duplicate (Fig. 1).

### Measurement of pH, titratable acidity, and viable cell counts

The pH of each yogurt sample was measured using a pH meter (UB-10, Denver, USA). The titratable acidity (lactic acid, %) values of each yogurt sample were determined by measuring the amount of 0.1 N NaOH necessary to adjust to pH 8.3. Viable cell counts were determined by plating on BCP agar plates with 20 uL of serial diluted cinnamon extracts after fermenting for 48 h. Each yogurt sample was stored in a cold room for 4 weeks.

### Sensory evaluation of yogurt products

The sensory evaluation of the yogurt supplemented with cinnamon ethanol extracts was performed by 10 trained panelists using randomly coded yogurt samples. The samples were provided to panelists at the same time. The color, flavor, taste, texture, and overall preference were determined using a 9-point hedonic scale (9 = extremely like, 8 = very much like, 7 = moderately like, 6 = slightly like, 5 = neither like nor dislike, 4 = slightly dislike, 3 = moderately dislike, 2 = very much dislike, and 1 = extremely dislike).

### Statistical methods

Data were expressed as mean ± SD (standard deviation), and statistical analysis for single comparisons was performed using Student’s *t*-test. Each experiment was repeated at least three times to yield comparable results. Values of *p* < 0.05 and *p* < 0.01 were considered significant.

## Results and discussion

### Antifungal activation of cinnamon extracts

**Fig. 2:**
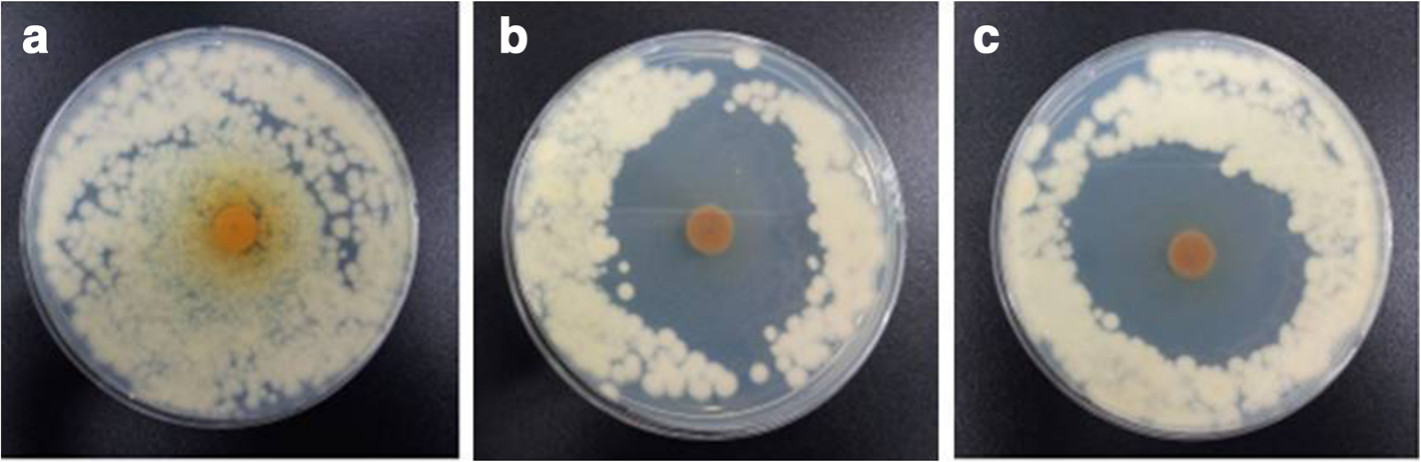
Inhibition zone photographs of cinnamon solvent extracts on the *Penicillium brevicompactum* strain FI02 growth. **a** cinnamon water extract; **b** cinnamon methanol extract; **c** cinnamon ethanol extract

The antifungal activity of cinnamon extracts was observed in treatments with either cinnamon ethanol extracts or cinnamon methanol extracts, but not in the treatment with hot water cinnamon extracts, as shown in Table 1 and Fig. 2. Specifically, the clear zone was 40 ± 1.0 mm wide in the treatment with cinnamon ethanol extracts, and 42 ± 1.1 mm wide in the treatment with cinnamon methanol extracts; therefore, the cinnamon methanol extracts had the highest antifungal effect. The reason for this result may be that the extraction yield of substances like cinnamaldehyde and eugenol, which are main components of *Cinnamomum cassia* bark, which account for its antifungal activity, is higher in solvents such as ethanol and methanol than in water [15, 16]. In comparison of the solvents, antifungal activity of methanol extracts was highest, but the effects of ethanol extracts were not significantly different, therefore ethanol extracts were used in this study because they can be used in food products like fermented milk. Min and et al. [17], who examined 231 kinds of materials including animal and plant crude drugs and natural plants regarding their antifungal activity, confirmed that *Cinnamomum cassia* bark exhibits prominent antifungal activity. Taken together, the use of cinnamon ethanol extracts may enable maximization of the antifungal activity without causing harm to the human body for application of cinnamon to food production for its antifungal activity.

**Table 1:** Antifungal activities of cinnamon extracts by different solvents

Strain	Inhibition zone diameter (mm)		
	Water	Methanol	Ethanol
*P. brevicompactum*	-	42 ± 1.1^a^	40 ± 1.0
strain FI02			

^a^All values are mean ± S.D

### Quality characteristics of fermented milk during production

**Fig. 3:**
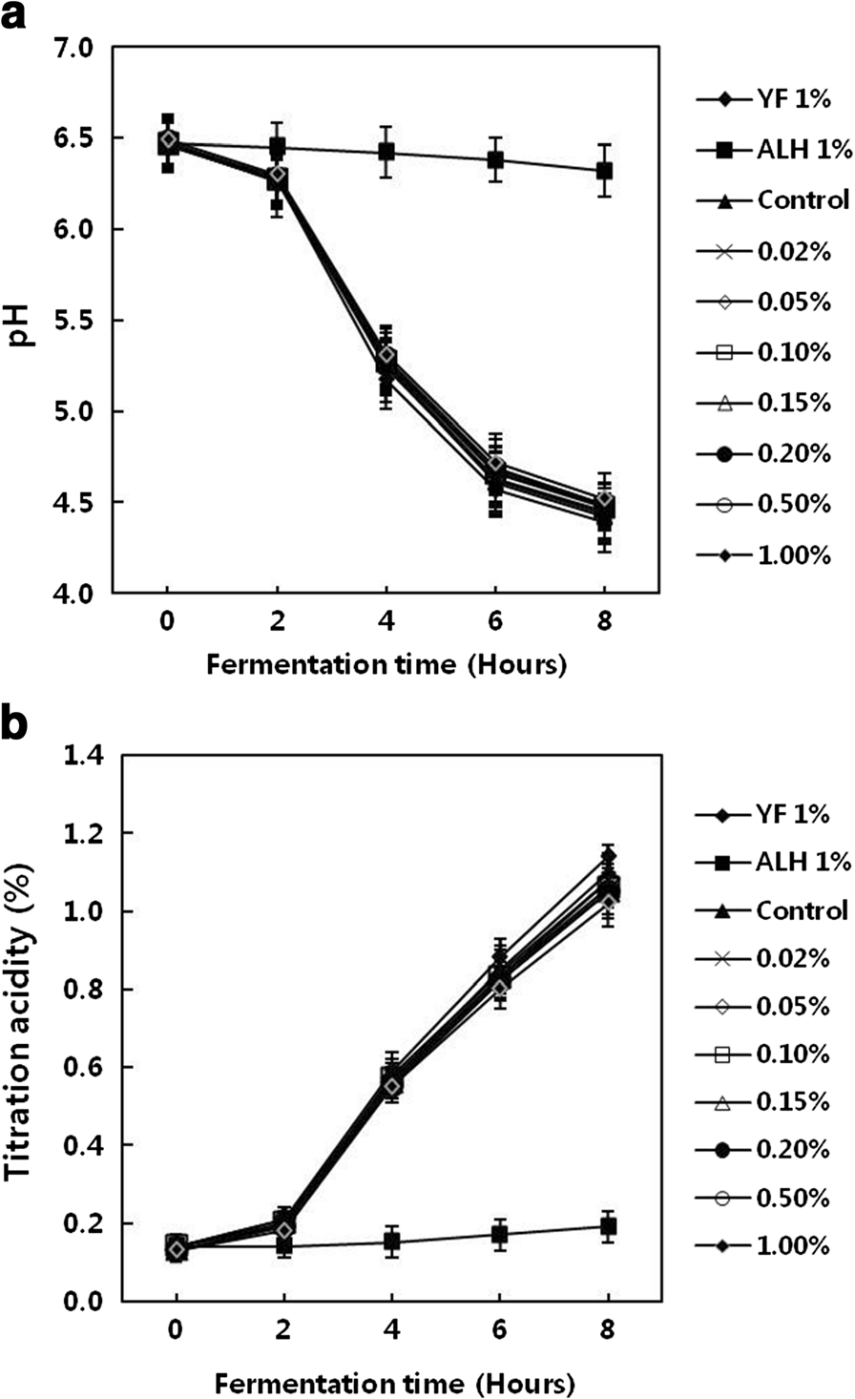
Changes in pH (**a**) and titration acidity (**b**) of yogurt made with lactic acid bacteria isolated from kimchi and cinnamon ethanol extract during fermentation at 40 °C for 8 h. YF 1 %, YF-L812 (*Lactobacillus delbrueckii* subsp. *bulgaricus*, *Streptococcus thermophilus*) 1.0 %; ALH 1 %, ALH (*Lactobacillus sakei* ALI033) 1.0 %; Control, YF-L812 0.3 % plus ALH (*Lactobacillus sakei* ALI033) 0.7 %; 0.02 %, Control plus Cinnnamon ethanol extract 0.02 %; 0.05 %, Control plus Cinnnamon ethanol extract 0.05 %; 0.10 %, Control plus Cinnnamon ethanol extract 0.10 %; 0.15 %, Control plus Cinnnamon ethanol extract 0.15 %; 0.20 %, Control plus Cinnnamon ethanol extract 0.20 %; 0.50 %, Control plus Cinnnamon ethanol extract 0.50 %; 1.00 %, Control plus Cinnnamon ethanol extract 1.00 %. All values are expressed as mean ± SD of triplicate determinations

To examine the synergy effect between ALH (*Lacobacillus sakei* ALI033) and cinnamon known as a natural antimicrobial, pH values of the mixture of fermented milk, ALH and cinnamon ethanol extract were measured over time and the results are shown in Fig. 3a. In the YF 1 %, control, treatments with less than 0.02 % cinnamon ethanol extracts and with less than 0.05 % extracts, it took 6 h to reach the fermentation endpoint, pH 4.6, while it took 7 h to reach it in the treatment with more than 0.10 % cinnamon ethanol extracts. These results indicate that the addition of cinnamon extracts to the extent of 0.05 % or lower concentration does not inhibit the metabolic activity of lactic acid bacteria in fermented milk. The measured values of titratable acidity over time are shown in Fig. 3b. After adding the cinnamon ethanol extracts, the values of titratable acidity continued to increase until the completion of fermentation. Bae et al. [18] reported in an identical manner that acidity continued to increase regardless of the addition of red ginseng extracts. The titratable acidity of normal fermented milk products is within the range of 0.7 ~ 1.2 % [19, 20], and the measured acidity in this study was 0.80 ~ 0.88 % after 6 h of fermentation, and 1.02 ~ 1.14 % after 8 h of fermentation; therefore, the fermentation is considered to have proceeded in a normal way. The measurements of the changes in the number of viable cells over time are shown in Table 2. Except in the treatment of 1.00 % with cinnamon ethanol extracts, the numbers of viable cells were stable at 4 × 10^9^ CFU/mL in all other treatments. This result might suggest that addition of more than 1.00 % cinnamon ethanol extracts to fermented milk can suppress the growth of viable cells because of cinnamaldehyde, a main component of *Cinnamomum cassia* bark. According to Korea’s processing criteria and component specification of livestock products (2010), the criterion for the total number of viable cells is 10^7^ CFU/mL and that for stirred yogurts is 10^8^ CFU/mL or more [21]. In this study, milk fermented for 6 h contained 10^9^ CFU/mL of viable cells, which is about 10 to 100 times more than the standard criterion, therefore it will have higher value in terms of product quality.

**Table 2:** Changes in viable cell counts of yogurt made with lactic acid bacteria isolated from kimchi and cinnamon ethanol extract during fermentation at 40 °C for 8 h

(CFU/mL)						
Ingredients		Fermentation time (h)				
		0	2	4	6	8
YF 1 %		5.3 × 10^6^	7.8 × 10^7^	6.3 × 10^8^	4.3 × 10^9^	2.3 × 10^10^
ALH 1 %		5.0 × 10^6^	6.2 × 10^6^	7.8 × 10^6^	8.3 × 10^6^	4.6 × 10^7^
Control		5.1 × 10^6^	7.6 × 10^7^	6.2 × 10^8^	4.2 × 10^9^	2.2 × 10^10^
Cinnnamon ethanol extract	0.02 %	5.1 × 10^6^	7.7 × 10^7^	6.1 × 10^8^	4.3 × 10^9^	2.1 × 10^10^
	0.05 %	5.0 × 10^6^	7.5 × 10^7^	6.0 × 10^8^	4.1 × 10^9^	2.0 × 10^10^
	0.10 %	5.1 × 10^6^	7.6 × 10^7^	6.1 × 10^8^	4.4 × 10^9^	2.3 × 10^10^
	0.15 %	5.0 × 10^6^	7.5 × 10^7^	6.1 × 10^8^	4.2 × 10^9^	2.3 × 10^10^
	0.20 %	5.0 × 10^6^	7.5 × 10^7^	6.2 × 10^8^	4.2 × 10^9^	2.2 × 10^10^
	0.50 %	5.0 × 10^6^	7.5 × 10^7^	6.0 × 10^8^	4.1 × 10^9^	2.0 × 10^10^
	1.00 %	4.9 × 10^6^	7.3 × 10^7^	5.9 × 10^8^	3.8 × 10^9^	1.8 × 10^10^

YF 1 % : YF-L812 (*Lactobacillus delbrueckii* subsp. *bulgaricus*, *Streptococcus thermophilus*) 1.0 %

ALH 1 % : ALH (*Lactobacillus sakei* ALI033) 1.0 %

Control : YF-L812 0.3 % + ALH (*Lactobacillus sakei* ALI033) 0.7 %

### Sensory evaluation of fermented milk

The results of sensory analysis of fermented milk with the mixture of ALH and cinnamon ethanol extracts are shown in Table 3. In the sensory evaluation, no significant differences in the category of texture and color were observed between the control and the treatment with cinnamon ethanol extracts, while the subjects showed higher preference for the treatment with less than 0.15 % extracts but lower preference for the treatment with more than 0.20 % in the odor category. The low acceptability of the control containing the mixture of commercial strains and ALH is thought to be due to the peculiar odor of kimchi during the ripening period. In the category of taste, the subjects showed very low preference for the treatment with more than 0.15 % cinnamon ethanol extracts due to the characteristic taste of cinnamon, which resulted in the accumulation of bitter taste according to the amount of cinnamon added. Therefore, the overall acceptability was highest in the yogurt containing 0.05 % cinnamon ethanol extract, in which the peculiar odor of kimchi is adequately neutralized by the flavor and taste of cinnamon. These results may be applied to the development of fermented milk products with excellent antifungal and antibacterial activity enhanced through the synergy between lactic acid bacteria isolated kimchi and cinnamon ethanol extracts without affecting the sensory characteristics of fermented milk.

**Table 3:** Sensory evaluations of yogurt made with lactic acid bacteria isolated from kimchi and cinnamon ethanol extract

Ingredients		Sensory evaluation				
		Color	Flavor	Taste	Texture	Overall acceptability
YF 1 %		6.4 ± 0.87^1)d^	7.0 ± 1.18^b2)^	6.9 ± 1.20^c^	6.9 ± 0.87^d^	6.8 ± 1.27^bc^
Control		6.5 ± 0.86^d^	6.8 ± 1.07^ab^	6.7 ± 1.16^c^	6.8 ± 0.71^d^	6.9 ± 1.32^bc^
Cinnnamon ethanol extract	0.02 %	6.5 ± 0.85^d^	7.0 ± 0.97^b^	6.8 ± 1.03^c^	6.8 ± 0.52^d^	7.2 ± 1.23^c^
	0.05 %	6.5 ± 0.82^d^	7.1 ± 0.85^bc^	7.2 ± 1.42^d^	6.8 ± 0.48^d^	7.7 ± 1.16^d^
	0.10 %	6.5 ± 0.81^d^	7.1 ± 1.42^bc^	7.0 ± 1.41^cd^	6.9 ± 0.88^d^	7.3 ± 1.15^cd^
	0.15 %	6.4 ± 0.84^d^	7.2 ± 1.22^c^	6.4 ± 1.35^bc^	6.8 ± 0.55^d^	6.7 ± 1.25^bc^
	0.20 %	6.3 ± 0.82^d^	6.8 ± 1.27^ab^	4.7 ± 1.17^b^	6.8 ± 0.67^d^	5.6 ± 0.87^b^
	0.50 %	6.3 ± 0.85^d^	6.6 ± 1.15^a^	3.8 ± 1.13^a^	6.7 ± 0.52^d^	4.5 ± 0.94^ab^
	1.00 %	6.3 ± 0.81^d^	6.7 ± 1.14^a^	3.2 ± 1.21^a^	6.8 ± 0.53^d^	3.3 ± 0.63^a^

YF 1 % : YF-L812 (*Lactobacillus delbrueckii* subsp. *bulgaricus*, *Streptococcus thermophilus*) 1.0 %

Control : YF-L812 0.3 % + ALH (*Lactobacillus sakei* ALI033) 0.7 %

^1)^All values are mean ± S.D

^2)^Mean ± SD with different superscript within a row are significantly different (*p* < 0.05) by Duncan’s multiple range test. a < b < c < d

### Quality characteristics of fermented milk during storage

**Fig. 4:**
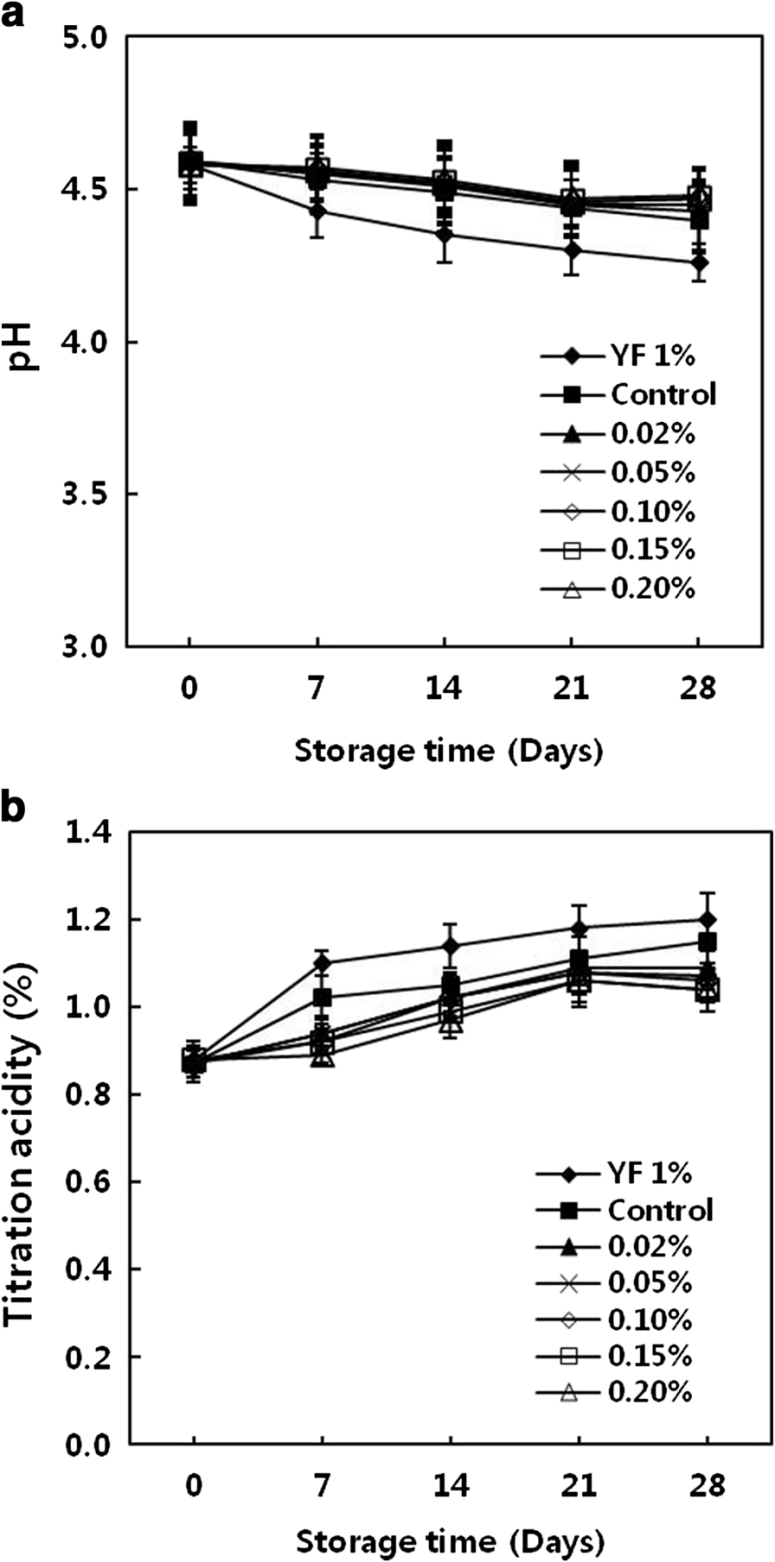
Changes in pH (**a**) and titration acidity (**b**) of yogurt made with lactic acid bacteria isolated from kimchi and cinnamon ethanol extract during storage at 4 °C for 28 days. YF 1 %, YF-L812 (*Lactobacillus delbrueckii* subsp. *bulgaricus*, *Streptococcus thermophilus*) 1.0 %; Control, YF-L812 0.3 % plus ALH (*Lactobacillus sakei* ALI033) 0.7 %; 0.02 %, Control plus Cinnnamon ethanol extract 0.02 %; 0.05 %, Control plus Cinnnamon ethanol extract 0.05 %; 0.10 %, Control plus Cinnnamon ethanol extract 0.10 %; 0.15 %, Control plus Cinnnamon ethanol extract 0.15 %; 0.20 %, Control plus Cinnnamon ethanol extract 0.20 %. All values are expressed as mean ± SD of triplicate determinations

Unlike other food products, fermented milk is stored and sold for a considerably long period at a low temperature, and the change in quality during storage is the major determinants in evaluating the products. We stored completely fermented milk in a refrigerator at 4 °C and measured pH, titratable acidity, and the changes in the number of lactic acid bacteria every 7 for 28 days. The results are shown in Fig. 4 and Table 4. In the YF 1 %, which was fermented only with the commercial starter, post acidification continued during the storage period, and pH value decreased by 0.32 from 4.58 to 4.26, titratable acidity increased by 0.32 % from 0.88 to 1.20. By comparison, in the control, which was fermented with the mixture of the commercial starter and lactic acid bacteria isolated from kimchi, pH value showed a decrease by 0.19 during the same period, and titratable acidity increased by just 0.28 %. In particular, in all treatments containing cinnamon ethanol extracts, the changes in pH and titratable acidity values were found to be much smaller compared with the control. Kim and et al. [22] reported that when the changes in pH and titratable acidity values were greater, the post acidification was accelerated and storage stability was reduced. This study confirmed that in fermented milk containing cinnamon ethanol extracts, the changes in titratable acidity were moderate and smaller compared with those in the control. This result suggests that the shelf life of fermented milk products by can be prolonged effectively suppressing post acidification using this method. In addition, the changes in the number of viable cells were smaller in the control and in all treatments with cinnamon extracts than in the YF 1 %, and the number continued to be over 10^8^ CFU/mL, which is more than the scope of optimal value, showing the stability of quality.

**Table 4:** Changes in viable cell counts of yogurt made with lactic acid bacteria isolated from kimchi and cinnamon ethanol extract during storage at 4 °C for 28 days

(CFU/mL)						
Ingredients		Storage time (days)				
		0	7	14	21	28
YF 1 %		4.3 × 10^9^	6.3 × 10^9^	9.3 × 10^9^	1.5 × 10^10^	3.2 × 10^10^
Control		4.4 × 10^9^	5.3 × 10^9^	7.6 × 10^9^	8.8 × 10^9^	9.8 × 10^9^
Cinnnamon ethanol extract	0.02 %	4.4 × 10^9^	5.2 × 10^9^	7.6 × 10^9^	8.8 × 10^9^	9.6 × 10^9^
	0.05 %	4.3 × 10^9^	5.3 × 10^9^	7.6 × 10^9^	8.7 × 10^9^	9.6 × 10^9^
	0.10 %	4.4 × 10^9^	5.1 × 10^9^	7.5 × 10^9^	8.7 × 10^9^	9.5 × 10^9^
	0.15 %	4.4 × 10^9^	5.2 × 10^9^	7.4 × 10^9^	8.6 × 10^9^	9.5 × 10^9^
	0.20 %	4.3 × 10^9^	5.2 × 10^9^	7.5 × 10^9^	8.7 × 10^9^	9.4 × 10^9^

YF 1 % : YF-L812 (*Lactobacillus delbrueckii* subsp. *bulgaricus*, *Streptococcus thermophilus*) 1.0 %

Control : YF-L812 0.3 % + ALH (*Lactobacillus sakei* ALI033) 0.7 %

## Conclusion

In conclusion, the results of this study suggest that the overgrowth of fermentation strains or post acidification can be delayed during storage, thereby maintaining the storage quality of yogurt products in a stable way, using *Cinnamomum cassia* bark, which exhibits excellent antifungal and antibacterial activity, in combination with lactic acid bacteria isolated from kimchi, which generate substances including organic acid, H_2_O_2_, and bacteriocin and thus produce antifungal and antibacterial activity.

## References

[1] Jang SS. Current status of fermented milk development. Korean J Food Sci Animal Resources. 2013;2:11–18.

[2] Domagala J (2009). Instrumental texture, syneresis and microstructure of yoghurts prepared from goat, cow and sheep milk. Int J Food Prop.

[3] Sah BNP, Vasiljevic T, McKechnie S, Donkor ON (2015). Effect of refrigerated storage on probiotic viability and the production and stability of antimutagenic and antioxidant peptides in yogurt supplemented with pineapple peel. J Dairy Sci.

[4] Leroy F, De Vuyst L (2004). Lactic acid bacteria as functional starter cultures for the food fermentation industry. Trends Food Sci Tech.

[5] Rhee S, Lee JE, Lee CH (2011). Importance of lactic acid bacteria inasian fermented foods. Microb Cell Fact.

[6] Luo YM, Luo YD, Chen FY, Liu H (2014). Studies on the chemical constituents in the essential oil from the leaves of cinnamomum Bodinieri Levi. Adv Mat Res.

[7] Son LC, Dai DN, Thang TD, Huyen DD, Ogunwande IA (2014). Study on cinnamomum oils: compositional pattern of seven species grown in Vietnam. J Oleo Sci.

[8] Buru AS, Pichika MR, Neela V, Mohandas K (2014). In vitro antibacterial effects of Cinnamomum extracts on common bacteria found in wound infections with emphasis on methicillin-resistant *Staphylococcus aureus*. J Ethnopharmacol.

[9] Udayaprakash NK, Ranjithkumar M, Deepa S, Sripriya N, Al-Arfaj AA, Bhuvaneswari S (2015). Antioxidant, free radical scavenging and GC-MS composition of Cinnamomum iners Reinw. ex Blume. Ind Crops Prod.

[10] Park UY, Kim SH, Kim JH, Kim YG, Chang DS (1995). Purification of antimicrobial substance for the extract from the root bark of Morus alba. J Food Hyg Safety.

[11] Cho EM, Bae JT, Pyo HB, Lee GS (2008). Antimicrobial plant extracts as alternative of chemical preservative: Preservative efficacy of *Terminalia chebula*, Rhus japonica(gallut) and cinnmomum cassia extract in the cosmetic formular. J Soc Cosmet Scientists Korea.

[12] Choi HN, Oh HH, Yang HS, Huh CK, Bae IH, Lee JS, Joeng YS, Joeng EJ, Jung HK. Antifungal activity against cheese fungi by lactic acid bacteria isolated from kimchi. Korean J Food Preserv. 2013;20:727–34.

[13] Yang EJ, Chang HC (2008). Antifungal activity of *Lactobacillus plantarum* isolated from Kimchi. Korean J Microbiol Biotechnol.

[14] Yilmaz MT, Dertli E, Toker OS, Tatlisu NB, Sagdic O, Arici M (2015). Effect of in situ exopolysaccharide production on physicochemical, rheological, sensory, and microstructural properties of the yogurt drink ayran: An optimization study based on fermentation kinetics. J Dairy Sci.

[15] Hill LE, Gomes C, Taylor TM (2013). Characterization of beta-cyclodextrin inclusion complexes containing essential oils (*trans*-cinnamaldehyde, eugenol, cinnamon bark, and clove bud extracts) for antimicrobial delivery applications. LWT-Food Sci Technol.

[16] Yen TB, Chang ST (2005). Synergistic effects of cinnamaldehyde in combination with eugenol against wood decay fungi. Bioresour Technol.

[17] Min BS, Bang KH, Lee JS, Bae KH (1996). Screening of the antifungal activity from natural products against *Candida albicans* and *Penicillium avellaneum*. Yahak Hoeji.

[18] Bae HC, Nam MS (2006). Properties of the mixed fermentation milk added with red ginseng extracts. Korean J Food Sci Anim Resour.

[19] Dello Staffolo M, Bertola N, Martino M, Bevilacqua A (2004). Influence of dietary fiber addition on sensory and rheological properties of yogurt. Int Dairy J.

[20] Ma C, Chen Z, Gong G, Huang L, Li S, Ma A (2015). Starter culture design to overcome phage infection during yogurt fermentation. Food Sci Biotechnol.

[21] Youm TH, Lim HB (2010). Antimicrobial activities of organic extracts from fruit of Thuja orientalis L. Korean J Med Crop Sci.

[22] Kim JK, Lee JS, Jeong YT, Bae IH (2012). Development of yoghurt with Sanmeoru(Vitis amurensis Ruprecht) wine as an additive. Korean J Dairy Sci Technol.

